# Use of Interferon Alfa in the Treatment of Myeloproliferative Neoplasms: Perspectives and Review of the Literature

**DOI:** 10.3390/cancers12071954

**Published:** 2020-07-18

**Authors:** Joan How, Gabriela Hobbs

**Affiliations:** 1 Department of Medical Oncology, Massachusetts General Hospital, Harvard Medical School, Boston, MA 02114, USA; chi-joan_how@dfci.harvard.edu; 2 Division of Hematology, Department of Medicine, Brigham and Women’s Hospital, Harvard Medical School, Boston, MA 02115, USA; 3 Department of Medical Oncology, Dana-Farber Cancer Institute, Harvard Medical School, Boston, MA 02115, USA

**Keywords:** interferon, pegylated interferon, ropeginterferon, myeloproliferative neoplasm, polycythemia vera, essential thrombocythemia, myelofibrosis

## Abstract

Interferon alfa was first used in the treatment of myeloproliferative neoplasms (MPNs) over 30 years ago. However, its initial use was hampered by its side effect profile and lack of official regulatory approval for MPN treatment. Recently, there has been renewed interest in the use of interferon in MPNs, given its potential disease-modifying effects, with associated molecular and histopathological responses. The development of pegylated formulations and, more recently, ropeginterferon alfa-2b has resulted in improved tolerability and further expansion of interferon’s use. We review the evolving clinical use of interferon in essential thrombocythemia (ET), polycythemia vera (PV), and myelofibrosis (MF). We discuss interferon’s place in MPN treatment in the context of the most recent clinical trial results evaluating interferon and its pegylated formulations, and its role in special populations such as young and pregnant MPN patients. Interferon has re-emerged as an important option in MPN patients, with future studies seeking to re-establish its place in the existing treatment algorithm for MPN, and potentially expanding its use for novel indications and combination therapies.

## 1. Introduction

Interferons are a group of cytokines that have a host of immunomodulatory, antiproliferative, and antiangiogenic properties, and were initially named for their ability to “interfere” with viral replication [1]. Human leukocyte interferons were first purified and studied in clinical trials in the late 1970s [2]. In the 1980s, cloning and large-scale production of interferon alfa allowed its evaluation in multiple oncologic diseases [3,4], such as multiple myeloma [5], hairy cell leukemia [6], chronic myeloid leukemia (CML) [7], and Philadelphia chromosome-negative myeloproliferative neoplasms (MPNs) including polycythemia vera (PV), essential thrombocythemia (ET), and myelofibrosis (MF) [8,9]. Currently, interferon alfa is approved in the treatment of hairy cell leukemia, hepatitis B and C infections, malignant melanoma, AIDS-related Kaposi sarcoma, follicular lymphoma and condyloma acuminata, and has additional off-label use for many hematologic and solid cancers including MPNs (Table 1). 

Early studies on the use of interferon alfa in MPNs demonstrated excellent hematologic responses, including normalization of platelet and leukocyte counts in ET and a reduction in phlebotomy in PV [8,9]. In vitro studies of interferon alfa treatment also showed a reduction in hematopoietic progenitor growth, with MPN clones showing greater sensitivity to interferon’s antiproliferative effects compared to normal progenitors [10,11,12]. In PV and ET patients, interferon alfa treatment was able to suppress clonal hematopoiesis, as demonstrated through early X-inactivation studies [13,14,15]. In CML, interferon represented the mainstay of treatment prior to the development of tyrosine kinase inhibitor (TKI) therapy and was the first agent able to induce sustained cytogenetic remissions [16]. 

However, despite its promising therapeutic properties, interferon alfa’s side effect profile and its lack of official regulatory approval were major factors in its disuse in recent decades. Only recently has interest in interferon alfa re-emerged. Reports that interferon alfa could lead to improvements in *JAK2 V617F* and *CALR* driver mutation burdens renewed further interest in its use for treatment of MPNs [17,18,19]. The development of more tolerable forms of interferon, including the pegylated form, has further expanded interest. 

The purpose of this review is to discuss interferon alfa’s current role in the treatment of Philadelphia chromosome-negative MPNs, including the use of more recent formulations such as pegylated interferon and ropeginterferon, and in special populations such as young and pregnant patients. 

## 2. Mechanism of Action of Interferon

The interferon family is categorized into three types, of which interferon alfa belongs to the Type I group and is broadly involved in the innate defense against viral infections [20]. Interferon alfa-2 has been the predominant Type I interferon used as a therapeutic agent, and includes standard interferon alfa-2b (Intron A), pegylated interferon alfa-2a (Pegasys), pegylated interferon alfa-2b (PegIntron), and, most recently, ropeginterferon alfa-2b (Besremi). 

Both Type I and II interferons exert their effects through human interferon alfa receptor chains, which activate signaling via the Janus kinase (JAK)-signal transducer and activator of transcription (STAT) pathway [21,22]. The anti-viral properties of interferon have led to its successful use in diseases such as hepatitis B and C and Kaposi sarcoma. Interferon’s mechanism of action in MPNs is less clear. Numerous studies have demonstrated an apoptotic effect on hematopoietic progenitors, with a preference for the mutated clone [23,24]. Colony unit megakaryocyte proliferation is also directly targeted, which likely accounts for interferon’s effects on thrombocytosis. In addition, there is evidence that interferon inhibits thrombopoietin (TPO) activation via suppression of JAK2 substrate phosphorylation [25]. Mouse models have shown that interferon can directly activate dormant hematopoietic stem cells in vivo [26], and specifically can induce differentiation in *JAK2 V617F* mutated quiescent stem cells [27]. This results in potential long-term depletion of the mutant clone via direct targeting of progenitor cells.

However, interferon also induces broader stimulatory effects on the immune system, enhancing surveillance and targeting of the mutant clone. Studies have demonstrated that interferon augments T-cell, macrophage, and natural killer cell activity, and increases expression of tumor-associated and major histocompatibility complex antigens [28,29,30]. These immunologic changes implicate immunotherapy as a potential therapeutic modality of MPN. Additional putative mechanisms of interferon include its pro-apoptotic properties, with gene expression profiles demonstrating induction of known mediators of apoptosis such as caspase and tumor necrosis factor-related apoptosis-inducing ligand (TRAIL) [31]. It is unclear how interferon’s anti-angiogenic effects contribute to its efficacy in myeloid malignancies [32], although it is possible that angiogenesis plays a role in the tumor microenvironment. 

## 3. Clinical Use of Interferon

### Essential Thrombocythemia and Polycythemia Vera

Although interferon is not currently approved for treatment in ET and PV, consensus guidelines recommend interferon as an option for first-line cytoreduction, and in particular in younger or pregnant patients (Table 1) [33,34]. Standard interferon alfa-2b has been used for decades in the treatment of ET and PV, with induction of hematologic—and in later clinical trials, molecular—remissions [8,9]. In one prospective, open-label, observational trial in 123 ET and 136 PV patients, interferon alfa demonstrated hematologic response rates of 73% and 71%, respectively [35]. This is similar to hematologic response rates with hydroxyurea [35]. Molecular responses, as defined by decreases in mutant allele burden by 20–50% (minor), greater than 50% (partial), or to undetectable levels (complete), also occurred in 55% of ET and 19% of PV patients with *JAK2 V617F* mutations [35]. There is no evidence that interferon can improve overall survival and alter the biologic disease course, such as reducing transformation to myelofibrosis or leukemia; however, interferon can reduce incidence of venous thromboembolism (VTE) in high-risk ET and PV patients [35,36,37]. 

Interferon alfa also has been reported to improve MPN-related symptoms, including a reduction in paresthesia and erythromelalgia in PV [8,9]. However, despite its clinical efficacy, interferon alfa is associated with a significant toxicity profile. Common side effects include flu-like symptoms, fever, malaise, nausea, and vomiting, with attrition rates of up to 35% reported in some clinical trials [8,9,38]. Interferon alfa is also dosed three times weekly, typically at 3 million units per dose. Given these limitations, pegylated forms of interferon (pegylated interferon alfa-2a and 2b) have been developed, which has improved tolerability and can be given once a week. Clinical trials of over 400 ET and PV patients have demonstrated overall hematologic remission rates of approximately 80% with pegylated interferon, including freedom from phlebotomy in 60% of PV patients [8,9]. Pegylated interferon is also associated with molecular response rates in 20–60% of *JAK2*-mutated patients [18]. Interestingly, the presence of concomitant non-driver mutations is associated with smaller mean decreases in *JAK2 V617F* allele burden with pegylated interferon treatment [39]. Interferon may have decreased ability to eradicate *TET2*-positive clones even with elimination of the *JAK2 V617F*-mutant clone [10], suggesting that the genomic landscape of MPN patients can predict responses to interferon treatment. Similarly, pegylated interferon alfa can induce hematologic and molecular responses in *CALR*-mutated ET patients [40]. In a cohort of 31 *CALR*-mutated ET patients, hematologic responses were achieved in all patients and median *CALR* variant allele fraction decreased from 41% to 26%, with two patients achieving complete molecular remission. Decreases in *CALR* allele fraction correlate closely with other measures of disease burden including lactate dehydrogenase, hemoglobin, and white blood cell and platelet counts [41]. Similar to what has been found in *JAK2 V617F*-mutated patients, the presence of additional mutations such as *TET2, ASXL1, IDH2*, and *TP53* was associated with poorer molecular responses [40]. There is evidence that molecular responses are more easily obtained in *JAK2 V617F* compared to *CALR* patients, thought to be due to increased interferon sensitivity of *JAK2 V617F*-mutant clones [42].

These responses are also durable, which is an appealing aspect of interferon treatment. In a long-term follow up of 83 ET and PV patients treated with pegylated interferon, median duration of hematologic and molecular response was 66 and 53 months, respectively [43]. In addition, three patients were able to sustain a complete molecular remission even after discontinuation of therapy, although in most patients *JAK2 V617* allele burden eventually increased after the first 24 months of treatment [43]. However, disease evolution remains similar to historical data in patients treated with other therapies [44], with a reported 8% transformation rate to myelofibrosis or leukemia [43]. VTE, the most common disease complication in ET and PV, occurred at a rate of 1.22 per 100 person-years, and is decreased compared to untreated high-risk ET and PV patients [43,45,46]. 

Recently, results from randomized trials comparing pegylated interferon head-to-head with hydroxyurea have been reported. MPN-RC-112 was a Phase III trial that randomized high-risk ET/PV patients to first-line treatment with either pegylated interferon alfa-2a or hydroxyurea (Table 2) [47,48]. The overall response rate of 78% with pegylated interferon was not significantly different from the overall response rate of 70% in hydroxyurea-treated patients. Best bone marrow responses occurred in 17% of evaluable patients treated with pegylated interferon, compared with 33% of patients treated with hydroxyurea (*p* = 0.052). Similarly, the Phase III DALIAH trial randomized 205 previously untreated MPN patients to interferon alfa-2a, interferon alfa-2b, or hydroxyurea, and interim analysis observed a 49% overall response rate in ET, PV, and pre-MF patients treated with interferon alfa, although this was significantly lower than the 75% response rate seen with hydroxyurea [49]. However, as pegylated interferon-treated patients often have continued clinical improvements over time, we await final analysis results with longer follow up. Partial molecular responses occurred in 16% of interferon-treated patients, compared to 23% in hydroxyurea-treated patients, with one complete molecular remission.

Pegylated interferon has also been studied specifically in ET and PV patients previously treated with hydroxyurea, a population that is associated with worse outcomes (Table 2) [51]. The Phase II single-arm MPD-RC-111 study evaluated pegylated interferon in 65 ET and 50 PV high-risk patients who were refractory to or intolerant of hydroxyurea [52]. Overall response rates at 12 months were 69% and 60%, respectively, in ET and PV patients, with improved responses seen in *CALR*-mutated patients. Complete response rates of 43% and 22% in ET and PV were lower compared to other studies, which can be attributed to the high-risk features of this study population. Bone marrow and molecular remissions were rare although follow up was shorter, with a median of 19.6 months. Arterial and venous thrombosis occurred in 2% and 5% of patients at 1 and 2 years, respectively, and 1 PV patient in the cohort progressed to myelofibrosis. 

Although pegylated interferon has improved tolerability compared to standard interferon, nearly all patients experience some adverse effects, with the most common being fatigue, myalgia, and nausea or vomiting and diarrhea. Neuropsychiatric side effects such as depression are also frequently encountered, with grade 3 symptoms occurring in 15% of ET and PV patients treated with pegylated interferon in one clinical trial [43]. Therefore, neuropsychiatric history should be considered prior to starting interferon, given the possibility of worsening symptoms. In addition, although toxicities improve with time, new late adverse effects including fatigue and depression occurred in 10–17% of patients annually after 24 months [43]. Laboratory adverse effects of pegylated interferon include hematologic abnormalities such as leukopenia, anemia, and thrombocytopenia, as well as elevation of liver function tests. Late autoimmune toxicities such as hypothyroidism, vasculitis, or hepatitis can also occur. When compared to hydroxyurea in MPD-RC-112 and DALIAH, grade 3/4 adverse events were significantly higher in pegylated interferon [47,49]. Discontinuation rates of pegylated interferon are still typically less than that of standard interferon, occurring in approximately 15–20% of patients on clinical trial [43,47,52], although in DALIAH, toxicity-dependent discontinuation was significantly increased in pegylated interferon (27%) compared to hydroxyurea (5%) [49]. Given that fatigue is also a common symptom in MPN patients, the non-trivial side effect profile of pegylated interferon makes it a less ideal choice for first-line treatment in patients who have significant disease-related symptom burden. 

Further efforts to improve interferon’s tolerability and administration have resulted in the recent development of ropeginterferon alfa-2b, which is a monopegylated interferon alfa given subcutaneously. Ropeginterferon’s extended half-life allows every other week dosing with extension to a monthly maintenance schedule. Its formulation as a home self-administered prefilled pen further improves patient compliance compared to pegylated interferon alfa-2a, the latter of which may pose unique challenges to patients such as dispensation of very small, difficult-to-measure volumes. A few recent trials have reported on the efficacy of ropeginterferon in PV patients (Table 3). The non-inferiority randomized Phase III PROUD-PV trial and its extension study CONTINUATION-PV evaluated ropeginterferon versus hydroxyurea in 257 PV patients with fewer than 3 years of prior cytoreductive treatment [53]. The primary endpoint was a composite outcome of hematologic response and spleen volume reduction. Given that few PV patients had enlarged spleens at enrollment, the trial failed to demonstrate the non-inferiority of ropeginterferon at 12 months. However, ropeginterferon was effective at inducing hematologic response, with 43% of patients experiencing complete hematologic response. The complete hematologic response rate in CONTINUATION-PV was significantly improved in ropeginterferon-treated patients compared to hydroxyurea-treated patients, and responses to ropeginterferon continued to increase over time. Molecular responses were not different between groups at 1 year (34% versus 42%), but were significantly improved in CONTINUATION-PV at 3 years (68% versus 33%). In addition to *JAK2 V617F*, ropeginterferon was also shown to reduce *TET2* allele burden, a mutation associated with inflammation that may contribute to disease acceleration in MPNs [53,54,55]. Ropeginterferon is also being evaluated in the low-risk PV population specifically, with interim analysis results of 100 patients recently reported for the LOW-PV study, a Phase II randomized trial comparing ropeginterferon with phlebotomy alone [56]. A total of 84% of ropeginterferon-treated patients met the composite endpoint of maintaining a hematocrit ≤ 45% for 12 months in the absence of progressive disease, compared to 60% of patients in the standard arm (*p* = 0.008), allowing the trial to stop early due to efficacy. This supports the use of ropeginterferon in the low-risk PV population as a means to reduce phlebotomy and maintain target hematocrit goals. 

Similarly, the Phase I/II study PEGINVERA evaluated efficacy and safety of ropeginterferon alfa in 51 PV patients regardless of prior cytoreductive therapy [57]. Patients who responded well following 1 year of biweekly treatment were able to switch to a more convenient monthly administration with maintenance scheduling, which occurred at a median of 2 years. Preliminary results reported that nearly all patients (41/42) had a hematologic response, with complete responses occurring in 64% of patients after a median of 34 weeks. Complete and partial molecular responses also occurred in 28.6% and 45.2% of all patients. Significantly, there was an over 80% complete hematologic and molecular response rate in patients who were able to switch over to monthly maintenance dosing, with 100% compliance. 

The most common adverse events reported with ropeginterferon include thrombocytopenia, leukopenia, increased liver function tests, arthralgia, flu-like symptoms and fatigue. The majority of toxicities were mild, and discontinuation rates in PROUD-PV and CONTINUATION-PV were 8% [53,58]. PEGINVERA utilized a maximum-tolerated dose design during the first year, resulting in discontinuation of ropeginterferon in 21 out of the 51 patients [57]. As only 8 of the 21 patients discontinued after the first year due to treatment-related adverse effects, the investigators recommended slower uptitration of ropeginterferon for better tolerability. LOW-PV also reported 6% grade ≥3 adverse events in ropeginterferon-treated patients, which was not significantly higher than standard therapy patients, again indicating its potential role in the low-risk PV population [56]. 

Given these favorable results, the Committee for Medicinal Products for Human Use (CHMP) has granted marketing authorization for ropeginterferon in Europe, and the Food and Drug Administration (FDA) has accepted a biologics license application for ropeginterferon’s approval in the United States. If approved, ropeginterferon would be the first interferon developed specifically for MPN. Ropeginterferon is also currently being evaluated in essential thrombocythemia (NCT 04285086).

## 4. Myelofibrosis

Interferon’s direct effects on clonal hematopoietic stem cells have generated interest in its use as a potential disease-modifying agent in MF. Several studies have demonstrated that both standard and pegylated interferon result in significantly decreased bone marrow fibrosis in a subset of MF patients with clinical response [58,59]. However, overall disease response has been disappointing [8,9]. In addition, toxicity, particularly hematologic toxicity with dose-limiting cytopenias, are more common in advanced MF compared to PV and ET, leading to more frequent discontinuation [8,9]. To date, allogeneic hematopoietic stem cell transplantation is the only known treatment that can modify the disease course in MF.

Studies evaluating interferon use in lower-risk MF patients have been more encouraging. A retrospective study of 62 MF patients treated with pegylated interferon demonstrated splenic response in 47%, constitutional symptom improvement in 82%, and resolution of leukocytosis and thrombocytosis in 60–80% of patients [60]. The majority of patients were low or intermediate risk, with only 21.3% of patients being high risk by International Prognostic Scoring System (IPSS). A prospective study of 30 Dynamic IPSS (DIPSS) low- or intermediate-1-risk MF patients demonstrated an overall response rate of 50% (including complete and partial remissions as well as clinical improvement) [61]. Forty percent of patients experienced a ≥50% decrease in spleen size, and of the 25 patients with available bone marrow biopsies, 5 showed a reduction in reticulin fibrosis. Patients without high-molecular-risk (HMR) mutations, including *ASXL1,* had significantly better responses. Interferon is recommended as an option in symptomatic low- or intermediate-risk MF patients by the National Comprehensive Cancer Network, although European Leukemia Net recommends consideration of hydroxyurea or ruxolitinib as first-line treatment in lower-risk MF patients requiring cytoreduction [33,62]. Ultimately, the decision for which cytoreductive agent to use in these patients is based on individual factors such as comorbidities, risk status, symptom burden, age, and toxicity profile. 

## 5. Combination Therapy

While interferons can induce remissions, not all patients respond, and side effects are frequently limiting. Combination therapy of interferon with JAK2 inhibitors such as ruxolitinib can potentially enhance interferon signaling, given that high levels of inflammation may be involved with resistance to interferon, and JAK2 inhibitors have anti-inflammatory properties [63]. Combination therapy may also permit lower doses, thus improving tolerability. The Phase II COMBI clinical trial evaluated ruxolitinib and pegylated interferon alfa-2a in 32 PV and 18 MF patients, of which 46 patients were intolerant of or refractory to pegylated interferon monotherapy [64]. There were no high-risk MF patients included in this trial, with the majority being low or intermediate-1 risk by DIPSS+. PV patients were included if they had evidence of active disease with need for cytoreduction. Thirty-one percent and forty-four percent of PV and MF patients achieved remission. The discontinuation rates were 6% for PV and 32% for MF patients, and overall symptom scores, as measured by MPN Symptom Assessment Forms (SAFs), decreased from 22 to 15. There was a significant decrease in *JAK2 V617F* allele burden at 2 years. Similarly, the phase I/II RUXOPEG trial evaluated ruxolitinib and pegylated interferon alfa-2a in treatment-naïve IPSS intermediate- or high-risk MF patients [65]. Investigators found significant decreases in spleen size and *JAK2 V617F* allele burden, as well as improvement in hematologic parameters in evaluable patients. These data suggest that combination therapy with ruxolitinib and interferon may prove to be an effective treatment with acceptable toxicity. Table 4 provides a summary of clinical trials evaluating pegylated interferon with ruxolitinib. 

## 6. Special Populations

### 6.1. Young Populations

Hydroxyurea is typically used as first-line cytoreduction in MPN patients. Although no difference has been found in secondary cancer rates between interferon and hydroxyurea [66], there are still concerns for hydroxyurea’s long-term effects [44,67,68]. Interferon’s potential for durable responses and disease modification also makes it a more appealing choice for younger patients, given that treatment can last for decades. Currently, expert consensus guidelines recommend consideration of interferon for first-line treatment in patients who are younger or who defer hydroxyurea [33,34]. 

### 6.2. Pregnant Populations

Although MPNs are typically a disease of older populations, up to 20% of patients are diagnosed before the age of 40 [69]. This introduces the specialized issue of pregnancy, with the majority of cases occurring in ET and very few occurring in MF. Pregnancy in MPNs is not contraindicated, with overall good outcomes and live birth rates of 70% in ET and PV [70,71]. However, MPN patients are at increased risk for pregnancy related complications, the most common being spontaneous abortion. Other common pregnancy related complications include higher risk for thrombosis and hemorrhage, pre-eclampsia, and stillbirths [70,72,73]. Cytoreduction is considered in patients who have higher risk features following guidelines for cytoreduction in MPN and may be given in those with prior pregnancy losses and/or complications, history of thrombosis, or extreme thrombocytosis leading to increased risk of bleeding [33,70,74].

The most commonly prescribed drug for cytoreduction in ET and PV patients, hydroxyurea, is a Category D drug in pregnancy, meaning that evidence of human risk to the fetus exists. Ruxolitinib is also contraindicated in pregnancy. Although interferons are a Category C drug, in which no controlled studies have been conducted in animals or humans, in general the literature has demonstrated safety of interferon for use in pregnancy [70,75]. Pharmacologic studies have not detected interferon in fetal blood or amniotic fluid [76]. Of 78 pregnancies treated with interferon reported in the literature, the live birth rate was 94%, with no fetal malformations [70]. Indeed, a meta-analysis showed improved live birth rates in ET women who were treated with interferon during their pregnancy [73]. Despite the association with improved outcomes, interferon use is typically reserved for high-risk pregnancies and it is unknown whether there would be any benefit in patients without traditional indication for cytoreduction.

There is less experience with pegylated interferon in pregnancy, although placental transfer of pegylated interferon is thought to be unlikely. Given its improved tolerability, it is reasonable for use instead of standard interferon in pregnant MPN patients. A case series of 10 ET pregnancies in 8 women starting treatment before conception with pegylated interferon reported 9 live births and 1 miscarriage, with no major bleeding or thrombosis events [77]. Current expert consensus guidelines recommend either interferon or pegylated interferon as first-line treatment when cytoreduction is needed in patients who are pregnant or considering pregnancy [33].

Interferons are present in breastmilk. However, the amounts are very low and it is unlikely that it will pose a significant risk in nursing mothers and breastfeeding infants [78]. Therefore, breastfeeding can be considered in women receiving interferon after delivery. 

## 7. Conclusions

Despite being a decades-old drug, interferon remains one of the only treatments with the potential for disease modification, given its impact on mutation burdens and induction of durable responses. However, its administration and toxicity profile were major barriers to its widespread use, resulting in the development of pegylated interferon and, more recently, ropeginterferon. Large meta-analyses of interferon’s effects are difficult due to different drug formulations and endpoints, but, overall, clinical trials have demonstrated clinical responses in the vast majority of ET and PV patients, as well as some molecular responses. Pegylated formulations have overall shown similar efficacy to hydroxyurea, and recent encouraging data surrounding ropeginterferon have led to ongoing efforts for official regulatory approval in MPNs. As a result, interferons are a reasonable cytoreductive alternative to hydroxyurea in ET and PV, and are recommended for first-line consideration in younger patients and pregnant women. Although interferon has limited efficacy in advanced MF, it does have use in lower-risk MF patients, with ability to improve symptoms, spleen size, and laboratory parameters. There is ongoing investigation on combination therapies with pegylated interferon and ruxolitinib in order to further improve efficacy and tolerability. The renewed interest in interferon will undoubtedly re-establish its place as an important component in the treatment of MPNs. 

## Figures and Tables

**Table 1:** FDA Approvals and Recommendations for Use of Standard Interferon, Pegylated Interferon, and Ropeginterferon in MPN.

FDA Approvals
Hairy cell leukemia Adjuvant treatment for high-risk melanoma ^1^ Clinically aggressive follicular lymphoma, with anthracycline-containing chemotherapy ^1^ AIDS-related Kaposi sarcoma Chronic hepatitis B Condylomata acumunata
Consensus Opinions on Use in MPN
Low-risk myelofibrosis Consider if symptomatic High-risk polycythemia vera and essential thrombocythemia: First-line treatment (as alternative to hydroxyurea) ^2^ Second-line treatment (if refractory to or intolerant of hydroxyurea) ^2^ Low-risk polycythemia vera and essential thrombocythemia Consider if symptomatic and need for cytoreduction Pregnant or breastfeeding MPN patients in need of cytoreduction

^1^ No longer clinically used. ^2^ National Comprehensive Cancer Network (NCCN) recommends consideration as first-line in young patients or patients considering pregnancy; European Leukemia Net (ELN) recommends interferon as acceptable first-line in high-risk polycythemia vera in need for cytoreduction.

**Table 2:** Summary of Clinical Trials Evaluating Single-Agent Pegylated Interferon Alfa 2A.

Clinical Trial	Phase	Patients	Comparator Arm	Clinical Response (RR/CR/PR)		Molecular Response (RR/CR/PR)		Discontinuation Rate	
				pIFN	Comparator	pIFN	HU	pIFN	Comparator
Masarova et al. 2017 [43]	2	ET (*n* = 40) and PV (*n* = 43)	None	80% ^A^/75%/5%		63%/18%/36%		22%	
DALIAH [49]	3	Untreated MPN (*n* = 205)	Hydroxyurea	49%/9%/33% ^A,B^	75%/19%/56% ^A,B^	17%/16%/1%	NR/23%/NR	27%	5%
MPD-RC 112 [48,50]	3	High-risk ET (*n* = 39) and PV (*n* = 43)	Hydroxyurea	78%/35%/43% ^C^	70%/37%/32% ^C^	11% ^D^/NR/NR	20% ^D^/NR/NR	15%	10%
MPN-RC 111 [47,48]	2	High-risk ET (*n* = 65) and PV (*n* = 50) resistant/refractory to hydroxyurea	None	65%/34%/31% ^C^		NR		14%	

^A^ Hematologic response; ^B^ ET, PV, and pre-MF patients only; ^C^ ELN response criteria; ^D^ cytogenetic response. CR: complete response; ET: essential thrombocythemia; MPN: myeloproliferative neoplasm; NR: not reported; PR: partial response; PV: polycythemia vera; pIFN: pegylated interferon; RR: response rate.

**Table 3:** Summary of Clinical trials Evaluating Ropeginterferon Alfa 2B.

Clinical Trial	Phase	Patients	Comparator Arm	Clinical Response (RR/CR/PR)		Molecular Response (RR/CR/PR)		Discontinuation Rate	
				rpIFN	Comparator	rpIFN	Comparator	rpIFN	Comparator
PEGINVERA [57]	1/2	PV (*n* = 51)	None	99%/64%/33% ^A^		74%/29%/45%		41%	
PROUD-PV [53]	3	PV with less than 3 years of cytoreduction (*n* = 257)	Hydroxyurea	21 ^B^/43% ^A^/NR	28% ^B^/46% ^A^/NR	34%/NR/NR	42%/NR/NR	NR	
CONTINUATION-PV [53]	3	PV with less than 3 years of cytoreduction (*n* = 257)	Hydroxyurea	53% ^C^/71% ^A^/NR	38%/51% ^A^/NR	66%/NR/NR	27%/NR/NR	8%	4%
LOW-PV [56]	2	Low-risk PV (*n* = 100)	Phlebotomy	84% ^D^/NR/NR	66%/NR/NR	NR	NR	NR	

^A^ Hematologic response; ^B^ Composite hematologic response and spleen size reduction; ^C^ Complete hematologic response and improvement in disease burden at 3 years; ^D^ Response Criteria: hematocrit < 45% with no progressive disease. CR: complete response; NR: not reported; PR: partial response; PV: polycythemia vera; rpIFN: ropegylated interferon; RR: response rate.

**Table 4:** Summary of Clinical Trials Evaluating Combination Pegylated Interferon Alfa 2A and Ruxolitinib.

Clinical Trial	Phase	Patients	Comparator Arm	Clinical Response (RR/CR/PR)		Molecular Response (RR/CR/PR)	Discontinuation Rate
RUXOPEG [65]	1/2	Intermediate/high-risk MF (*n* = 15)	None	0%/NR/NR ^A^	30%/NR/NR ^A^	NR	NR
COMBI [64]	2	PV (*n* = 32) and MF (*n* = 18) with active disease	None	31%/9%/22% (PV) ^B^ 44%/28%/72% (MF) ^A^		41%/2%/35%	6% (PV)32% (MF)

^A^ IWG-MRT Clinical response criteria; ^B^ ELN response criteria. CR: complete response; MF: myelofibrosis; NR: not reported; PR: partial response; PV: polycythemia vera; pIFN: pegylated interferon; RR: response rate.
